# Preserving osteopathic antiquity through historical pamphlets and postcards*

**DOI:** 10.5195/jmla.2020.876

**Published:** 2020-01-01

**Authors:** Lori Ann Fitterling, Robyn Oro

**Affiliations:** University Library Director, D’Angelo Library, Kansas City University of Medicine and Biosciences, Kansas City, MO, lfitterling@kcumb.edu; Access Services/Special Collections Coordinator, D’Angelo Library, Kansas City University of Medicine and Biosciences, Kansas City, MO, roro@kcumb.edu

## Abstract

During the late nineteenth and early twentieth centuries, osteopathic information was circulated by way of pamphlets and postcards. Several osteopathic historical pamphlets and postcards from the D’Angelo Library collection have been researched and digitized in order to preserve these osteopathic artifacts and highlight their historical significance for the current profession.

Although osteopathy has received little recognition in mainstream medical history, its proven 145-year legacy has garnered much support for osteopathic philosophies and treatments, and it is today an active branch of American medical practice. There are approximately 114,000 fully licensed practicing doctors of osteopathic medicine (DOs), and 25% of US medical students are training to be osteopathic physicians in 56 teaching locations in 33 states [1].

Early nineteenth century osteopathic philosophies, such as shifting the treatment of medical conditions away from prescription medicine and focusing on utilizing a whole body approach to treatment, are widespread in current health care practices. Publications of pamphlets and postcards from these early years showcase fundamental osteopathic principles and provide historical references about the practice of osteopathic physicians and early osteopathic hospitals, infirmaries, and sanitariums. Preservation of these physical artifacts extends the collective record of medical history and lays the foundation for current osteopathic medical practices.

Beginning as a reformation movement in search of an alternative to standard medical practice, osteopathy claims a formal beginning in 1874 with Andrew Taylor Still, MD, DO. A frontier physician known as the “Father of Osteopathy,” Dr. Still served an apprenticeship under his father and referred to himself as a licensed frontier physician (MD) and went on to establish the American School of Osteopathy in Kirksville, Missouri. Although he published several books—his autobiography (1897), *The Philosophy of Osteopathy* (1899), and *The Philosophy and Mechanical Principles of Osteopathy* (1902) [2–4]—it was not until 1906 that a preliminary history of the movement, the *History of Osteopathy and Twentieth-Century Medical Practice,* was formally published by Emmons Rutledge Booth, DO [5]. In 1924, Dr. Booth published a revised and expanded edition in which he updated information about osteopathic colleges and provided the first listing of osteopathic infirmaries and hospitals [6].

In these early years, osteopathic information was circulated by way of pamphlets, leaflets, and brochures. The archives in the Kansas City University of Medicine and Biosciences D’Angelo Library contain pamphlets and postcards that predate or are contemporary to the first published osteopathic medical history. These early publications have been designed with striking illustrations and photographs and describe new (at the time) medical theories such as the interrelated nature of bodily systems and musculoskeletal treatment techniques (e.g., osteopathic manual manipulation), with an overall emphasis on wellness and disease prevention.

The authors’ library began a robust preservation project that includes researching, cataloging, and digitizing osteopathic pamphlets and vintage postcards to make this collection accessible. This project provides a digital background for the historical significance of osteopathy, which has shaped the present approach of osteopathic patient care.

## PAMPHLETS

**Figure 1:**
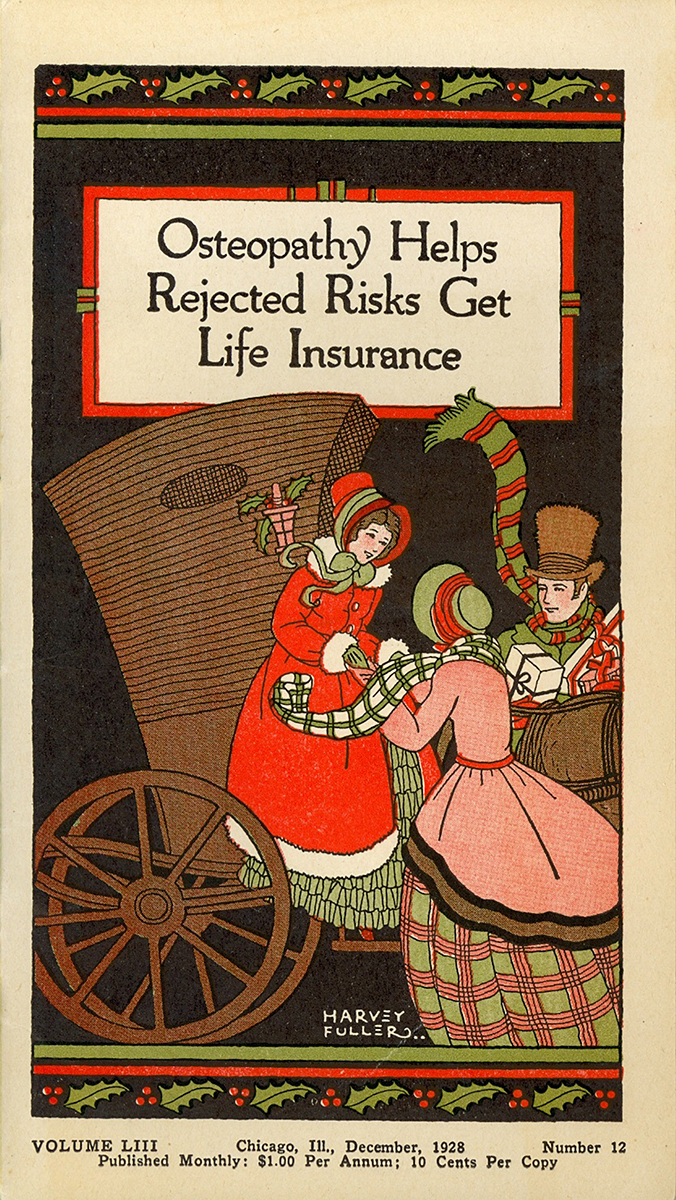
Osteopathy Helps Rejected Risks Get Life Insurance This 1928 pamphlet, published by Henry Stanhope Bunting, DO, an early promoter of osteopathy, and illustrated by Harvey Fuller, a popular illustrator of children’s books, served as a patient handout, explaining the principles and philosophies of osteopathy to the general public.

Twenty-three eye-catching pamphlets on osteopathy in the collection, many with color illustrations by the comic and children’s book illustrator, Harvey Fuller (Figure 1), were produced by three publishers: R. H. Williams, DO (Williams Publishing), nicknamed “antiseptic” Williams and a noted Kansas City DO [7]; Henry Stanhope Bunting, DO (Bunting Publishing), who went to school to become a DO after writing a story for a Chicago newspaper about A. T. Still [8]; and the American Osteopathic Association. Marketed as literature that would encourage and inform about osteopathy, the pamphlets could be considered a precursor to newspapers, periodicals, and patient handouts.

**Figure 2:**
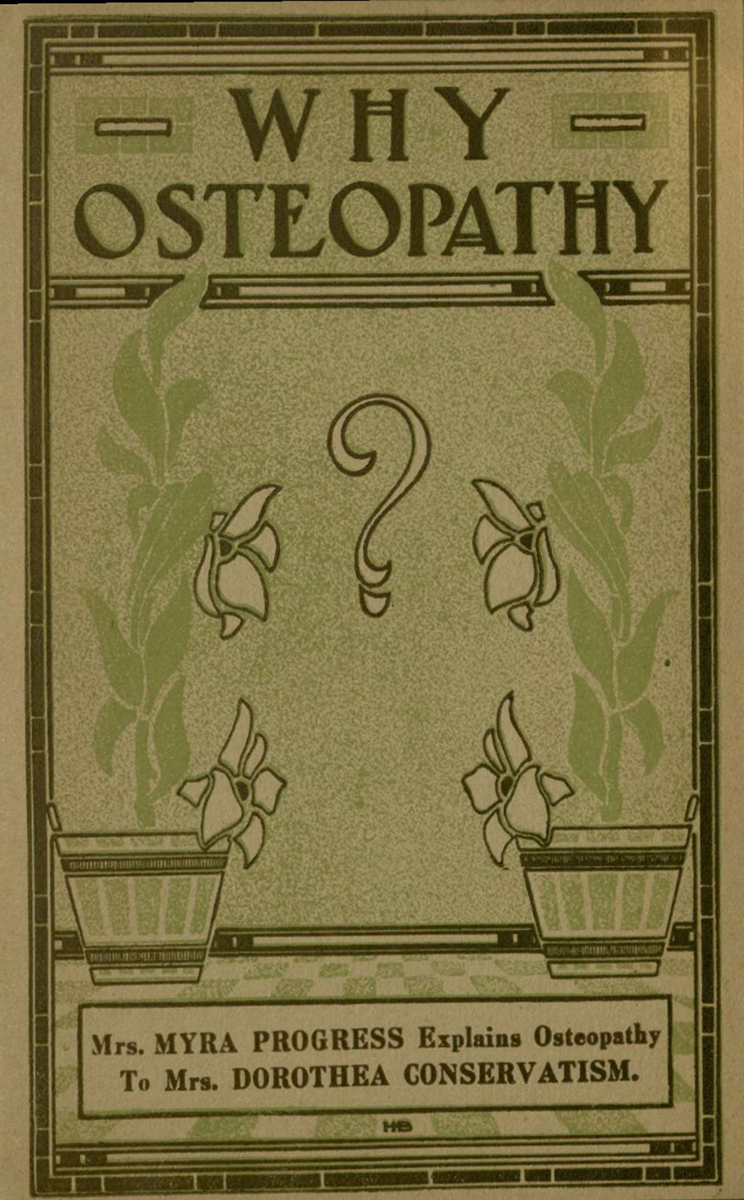
Why Osteopathy This 1912 pamphlet published by Kansas City osteopath and developer of antiseptic creams, R. H. Williams, DO, has Mrs. Myra Progress explaining osteopathy to Mrs. Dorothea Conservatism. 1910 pamphlet published

**Figure 3:**
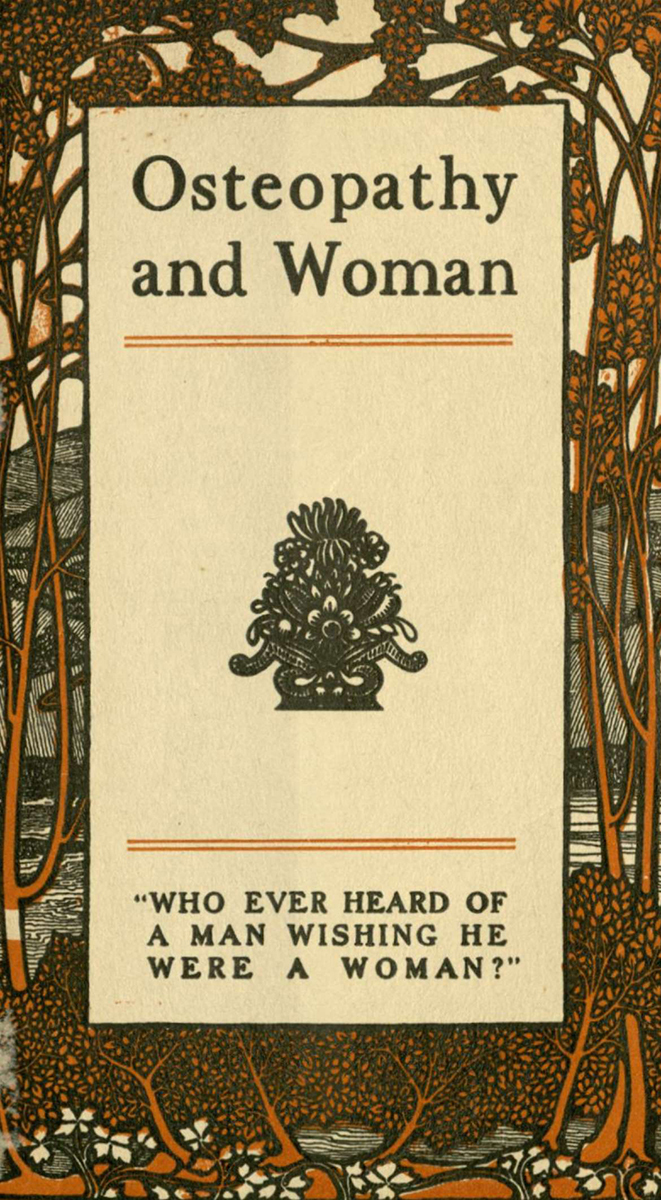
Osteopathy and Woman The 1910 pamphlet published by R. H. Williams, DO, titled *Osteopathy and Woman* and subtitled *Who Ever Heard of a Man Wishing He Were a Woman,* examines how osteopathic treatment “places [a] woman in a physical condition to take advantage of her improved social and industrial position, and do her best work in the world.”

Many of the pamphlets’ subjects focus on the merits of the broad scope of osteopathy (Figures 1–3). One pamphlet, *Health at PAR Osteopathy for the Business Man* (Bunting Publications; 1924) by Howard T. Treleaven, DO, is an example of how the osteopathic principle of maintaining one’s health was regarded [9]. After some admonition about keeping one’s body on par, Dr. Treleaven explains:

The field of Osteopathy is limited only by those comparatively few diseases in which the pathologic destruction of the tissues has gone so far that regeneration is physically impossible and for which there is no known cure. Its practice actually includes all of mankind’s ailments which the general practitioner of any system is called on to treat. It is common sense, combined with the application of physiologic laws, in the correction and prevention of abnormal influences which cause human illness and suffering. [9]

This “common sense” knowledge of the early osteopathic emphasis on prevention of human illnesses resonates widely in current patient care.

From another of the pamphlets, *Chats on Osteopathy* (1927), by Carl P. McConnell, DO [10]:

Thus we come back to the underlying principles of osteopathy, whereby structural conditions may be impaired through deleterious habits, bad hygiene, poor sanitation, overwork and fatigue, an unbalanced dietary, emotional strain and stress, and discordant environing forces. [10]

This type of essay, which emphasizes an archetypical osteopathic viewpoint, is found throughout these materials and is significant in recognizing that osteopathic philosophy could be considered visionary in promoting a more holistic approach to health and linking lifestyle factors to medical conditions.

Dr. Stanhope Bunting became a pioneer for disseminating early osteopathic information. He became impressed with Still’s movement after travelling to Kirksville, Missouri, to write a story for a Chicago newspaper in the mid-1890s. He eventually enrolled in the school and graduated in 1900, setting up a practice in Chicago and beginning two monthly publications, *Osteopathic Physician* for the practitioner only and *Osteopathic Health* for the general public [8]. These publications document historical philosophies and practices of osteopathic medicine.

Distributing these types of publications could be challenging. Norman Gevitz, in his book *The DOs: Osteopathic Medicine in America* [8]*,* describes how a DO would send the “actual or potential” names and addresses of patients to Dr. Stanhope Bunting, who would then, for a fee, send a one-year subscription of *Osteopathic Health* along with a business card of the DO [8]. This is one way that osteopathic medical information was transferred.

Some, but not all, pamphlets have prices on the covers (e.g., “$1.00 Per Annum; 10 Cents per Copy”) and list authors. One of Dr. Williams’s pamphlet states, “Paper read by Walter Anson Merkley, AB, DO, before the Rainy Day Club of New York, at the Hotel Astor” [11], which illustrates the dual nature of some of the pamphlets as not only written treatises, but verbal presentations. Other pamphlets published by Dr. Stanhope Bunting listed as many as 7 authors, and some include reprints from various sources such as the 1922 *Encyclopedia Britannica*, the *Journal of Osteopathy*, and the *Ladies’ Home Journal.*

For complete list of pamphlets, see the Kansas City University (KCU) Special Collections and Archival Material.

## POSTCARDS

The postcard collection includes various osteopathic hospitals, sanitariums, bath houses, historical sites, and college buildings. In general, the sending of postcards began in the United States in the late-1800s with the purpose of delivering short messages or pictures for a lower postage cost. Of the thirty-two postcards spanning the years 1908 to 2010 in the collection, eighteen relate directly to osteopathic history. Some of the postcards have handwritten notes on the back and are addressed with postage stamps and postmarked dates. Both sides of the postcards were scanned in the digitization process for preservation.

**Figure 4:**
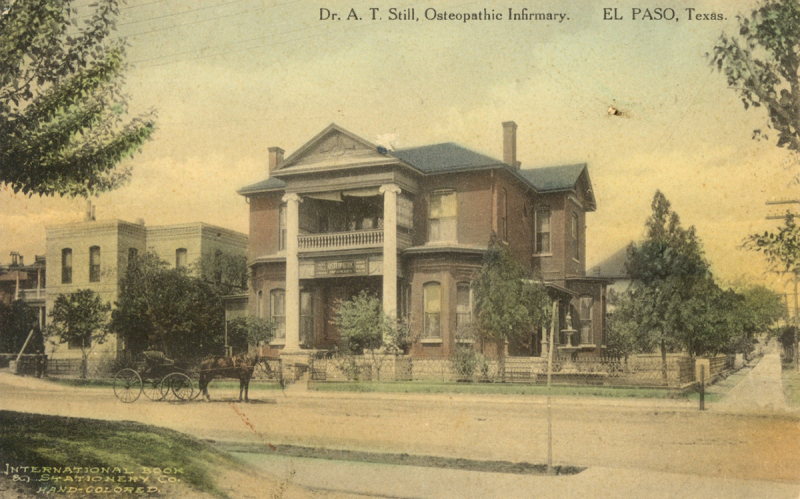
Dr. A. T. Still Osteopathic Infirmary This hand-tinted postcard depicts the A. T. Still Osteopathic (ASO) Infirmary that opened in 1904 in El Paso, Texas, by Ira W. Collins, DO, a new graduate of the American School of Osteopathy along with Dr. H. T. Still, one of A. T. Still’s sons. Dr. Collins claimed it was a branch of the ASO Infirmary in Kirksville, Missouri, the first health care facility in a school of osteopathy.

Several postcards in this collection showcase sanitariums and hospitals that had been converted from private homes, a common practice at this time [12]. In 1918, the American Medical Association denied DOs admitting or staff privileges in allopathic hospitals, and osteopaths were forced to build their own stand-alone hospitals. Funds from the Hill-Burton Act, passed in 1946, contributed to a boom of hospital construction, and between 1936 and 1950, as many as 374 hospitals were registered or approved by the American Osteopathic Association [12]. Changes in the health care marketplace as well as greater acceptance of osteopathic medicine and its integration with allopathic medicine toward the end of the 20th century led to the closing of many osteopathic hospitals. Many of those structures are gone today, but postcards have preserved pictorial evidence of this parallel American medical movement (Figure 4).

**Figure 5:**
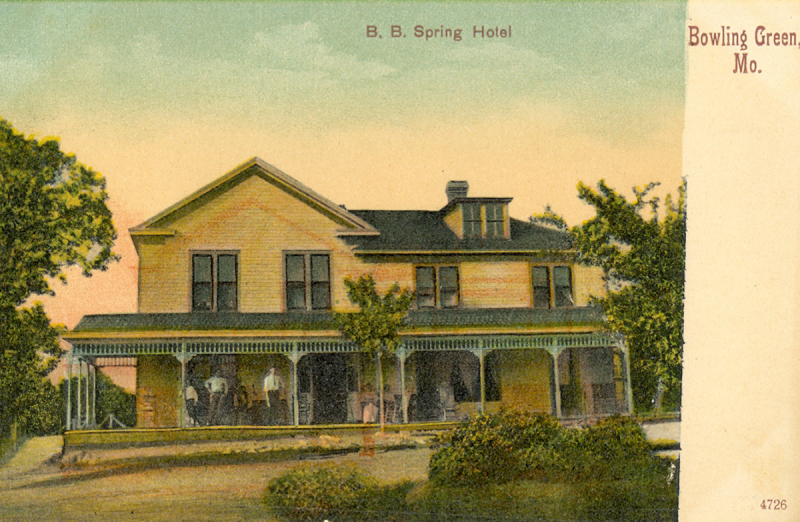
B. B. Spring Hotel Hand-tinted postcard depicting the B. B. Spring Hotel in Bowling Green, Missouri. The property would be purchased by R. H. Williams, DO, in 1923 and operated as the Bowling Green Sanitarium and Mineral Water Company. This card is of the Undivided Back Era (1901–1907) and was printed in Germany as were most postcards of this time.

One tinted postcard featuring the B. B. Springs Hotel in Bowling Green, Missouri (Figure 5), is among the oldest postcards of the collection and shows people standing and sitting on the front porch. The hotel was purchased by three osteopaths— R. H. Williams, DO, C. H. Downing, DO, and H. S. Hain, DO—and opened in 1923 as the Bowling Green Sanitarium and Mineral Water Company, specializing in osteopathic and hydrotherapy. Dr. Williams also marketed Bowling Green Mineral Water.

Another sepia printed postcard displays an image of the Southwestern Osteopathic Sanitarium in Blackwell, Oklahoma. The sanitarium was established in 1912 and moved to the building pictured on the postcard in 1915. George J. Conley, DO, was president of the Board of Control and surgeon-in-chief from 1914 to 1924. Dr. Conley would later be one of the founders and the first president of the Kansas City College of Osteopathy and Surgery (now the Kansas City University of Medicine and Biosciences). In 1924, the institution moved to Wichita, Kansas.

For complete list of the postcard collection, see the KCU D’Angelo Library Archives Collection.

## CONCLUSION

While the digital preservation of osteopathic historical collections ensures access for future generations, it also allows a wider audience to glimpse into the minds of these early promoters of A. T. Still’s movement. We are in great debt to their resolve to inspire the profession, and by preserving these historical pamphlets and postcards, we can better understand the osteopathic approach to patient care today.

The entire collection of osteopathic pamphlets and postcards can be viewed at the KCU Special Collections and Archival Material (pamphlets) and the D’Angelo Library Archives Postcard Collection (postcards).

## 

**Lori Ann Fitterling, MLS,** lfitterling@kcumb.edu, https://orcid.org/0000-0003-1614-6162, University Library Director, D’Angelo Library, Kansas City University of Medicine and Biosciences, Kansas City, MO

**Robyn Oro,** roro@kcumb.edu, Access Services/Special Collections Coordinator, D’Angelo Library, Kansas City University of Medicine and Biosciences, Kansas City, MO
